# Fish Oil Diet Associated with Acute Reperfusion Related Hemorrhage, and with Reduced Stroke-Related Sickness Behaviors and Motor Impairment

**DOI:** 10.3389/fneur.2014.00014

**Published:** 2014-02-06

**Authors:** Michaela C. Pascoe, David W. Howells, David P. Crewther, Nicki Constantinou, Leeanne M. Carey, Sarah S. Rewell, Giovanni M. Turchini, Gunveen Kaur, Sheila G. Crewther

**Affiliations:** ^1^Brain Sciences Institute, Swinburne University, Melbourne, VIC, Australia; ^2^Florey Neuroscience Institutes, Melbourne, VIC, Australia; ^3^School of Psychology, Deakin University, Geelong, VIC, Australia; ^4^School of Life and Environmental Sciences, Deakin University, Warrnambool, VIC, Australia; ^5^Institute of Sport Exercise and Active Living, Victoria University, Melbourne, VIC, Australia; ^6^School of Psychological Science, La Trobe University, Melbourne, VIC, Australia

**Keywords:** polyunsaturated fatty acids, middle-cerebral-artery-occlusion, mood, stroke

## Abstract

Ischemic stroke is associated with motor impairment and increased incidence of affective disorders such as anxiety/clinical depression. In non-stroke populations, successful management of such disorders and symptoms has been reported following diet supplementation with long chain omega-3-polyunsaturated-fatty-acids (PUFAs). However, the potential protective effects of PUFA supplementation on affective behaviors after experimentally induced stroke and sham surgery have not been examined previously. This study investigated the behavioral effects of PUFA supplementation over a 6-week period following either middle cerebral artery occlusion or sham surgery in the hooded-Wistar rat. The PUFA diet supplied during the acclimation period prior to surgery was found to be associated with an increased risk of acute hemorrhage following the reperfusion component of the surgery. In surviving animals, PUFA supplementation did not influence infarct size as determined 6 weeks after surgery, but did decrease omega-6-fatty-acid levels, moderate sickness behaviors, acute motor impairment, and longer-term locomotor hyperactivity and depression/anxiety-like behavior.

## Introduction

Ischemic stroke affects 15 million people a year and is a leading cause of disability worldwide (1). Recovery after stroke is associated with high incidences of behavioral and affective disorders including anxiety and (2, 3) clinical depression (4), which are commonly co-morbid (5, 6). In non-stroke affected populations, depressive disorder is associated with delirium (7, 8). Delirium is also common following stroke (9). Anxiety, depression, and delirium all negatively influence recovery after stroke (9, 10). Depressive disorder is also similarly closely linked to inflammation related “sickness behaviors” (11). Sickness behaviors are defined as the normal, ubiquitous responses to infection seen in all animal species examined and bear close biological resemblance to clinical depression (11). Sickness behaviors usually suppress normal activities such as eating and drinking. Thus to achieve the best prognostic outcome for stroke survivors, it is important to design better management regimes for post-stroke affective disorders (4) and sickness behaviors in general.

An alternative therapy for the generic treatment of depression (12), anxiety (13), and hyperactive symptoms (14) are long chain omega-3 polyunsaturated fatty acids (*n*-3-LC-PUFA) and in particular eicosapentaenoic acid (EPA; 20:5*n*-3) and docosahexaenoic acid (DHA; 22:6*n*-3) (14). Many studies, however, regarding the effectiveness of *n*-3-LC-PUFA in the treatment of emotional and behavioral disorders have found inconsistent results, and the number of studies is limited, indicating a need for additional research (15). The influence of such *n*-3-LC-PUFA supplementation on mood and behavioral disorders has not previously been investigated after stroke either in humans or in animals after experimentally induced ischemia via middle cerebral artery occlusion (MCAo).

*n*-3-LC-PUFAs are highly bioactive compounds, commonly derived from dietary fish oil, and have anti-inflammatory effects at optimal doses (12). *n*-3-LC-PUFAs have anti-thrombotic effects (16) and contribute to reduced platelet coagulation (17). Accordingly, some animal studies demonstrate that *n*-3-LC-PUFAs increase bleeding after experimentally induced intracerebral hemorrhage (18). We are unaware of any research indicating a potential risk of PUFA supplementation on bleeding after cerebral ischemia. Conversely, a number of previous studies have demonstrated neuroprotective effects of *n*-3-LC-PUFAs after cerebral ischemia in animal models (19–21). Thus, in the present study, we compared the effects of diet (*n*-3-LC-PUFA supplementation to the basal diet vs. basal diet) over the 6-weeks after surgery (either MCAo or sham surgery) in rats. In particular we aimed to study the effects of diet on surgically induced acute and longer-term motor and sickness behaviors, including behaviors commonly interpreted to reflect excessive anxiety or depressive-like behaviors in the rodent model.

We hypothesized that following MCAo surgery animals would show reduced food and water consumption, compared to shams. Additionally, we hypothesized that the *n*-3-LC-PUFA diet supplemented animals would show less reduction in food and water consumption, and body weight post-surgery, compared to the basal diet fed animals.

Acute inabilities to make co-ordinated motor movements have regularly been reported in rats post MCAo (22, 23) and decrease in response to neuroprotective agents (24–26). Thus, we hypothesized that the *n*-3-LC-PUFA supplemented animals would also show less acute MCAo related motor impairments on a battery of widely used neurological impairment tests (22, 23) compared to basal diet fed rats.

We hypothesized that hyperactive locomotor behaviors assessed using the free exploration test, would not correlate with anxiety-like behavior in this test, defined by percentage of emergence time spent in the center of the open-field arena. Previous authors have speculated that MCAo related changes in locomotion might arise from an inability to habituate to unfamiliar environments, possibly related to spatial mapping difficulties induced by hippocampus cell death (27–30). Therefore, spatial mapping abilities were assessed here using the spatial displacement recognition test, which has previously been employed to study spatial memory deficits post-stroke in the rodent model (28, 31–33).

We hypothesized that MCAo operated animals would show more long-term stroke-related anxiety-like and hyperactive locomotor behaviors than sham operated animals (MCAo vs. Sham). Finally, we hypothesized that after surgery animals supplemented with *n*-3-LC-PUFA would show less long-term sickness or anxiety-like and locomotor behaviors than basal diet fed animals (*n*-3-LC-PUFA vs. Basal). Long-term was defined as occurring at 2, 4, or 6 weeks following surgery.

In summary, we anticipated that *n*-3-LC-PUFA diet supplementation would reduce acute MCAo related motor impairments, and sickness behaviors. We expected the beneficial effects of *n*-3-LC-PUFA diet supplementation to persist longer term, as evidenced by a longer-term reduction in rodent behaviors, interpreted to reflect stroke-related behavioral and affective disorders.

## Materials and Methods

### Animals and design

The experiment used two surgery groups (MCAo and Sham groups) and two diet groups (basal and *n*-3-LC-PUFA supplementation) with repeated measures outcomes. Adult male hooded-Wistar rats (Laboratory Animal Services, The University of Adelaide, Australia) commonly used in stroke research (34–36) were separately housed (21 ± 2°C, 12:12 h light–dark cycle). Food and water were available *ad libitum* and consumption and body weight were measured daily. Acclimatization to housing and diet condition began 1 week prior to surgery and continued until animal sacrifice (via isoflurane anesthesia; Cat No. AHN3640-250ML, Baxter, Old Toongabbie, NSW, Australia) at 6 weeks post-surgery. Animal behavior was studied until 6 weeks post-surgery as previous research, in non-stroke-related areas, has demonstrated that 6 weeks of oral supplementation with omega-3 is associated with anti-depressive-like behavioral effects in rodent models (37). In the week following surgery only, animals were provided with sunflower seeds and soft food (Sustagen^®^ Everyday Nestlé, Notting Hill, VIC, Australia) additional to their respective diets, to encourage eating and weight gain. Surgeries and behavioral testing took place in the lights-on phase. The experimental protocol required 48 animals;j however 11 rats in the *n*-3-LC-PUFA MCAo group died immediately following withdrawal of the suture thread. A further 16 rats (*n*-3-LC-PUFA MCAo, *n* = 7; Basal MCAo, *n* = 9) were culled due to MCAo induced motor impairments and clinical symptoms deemed too large for the animal to survive. These animals were replaced and thus, the total number of animals used in the present study was 75 (*n* = 29 *n*-3-LC-PUFA MCAo; *n* = 22 Basal MCAo; *n* = 12 *n*-3-LC-PUFA sham; *n* = 12 Basal Sham).

### Ethics statement

Ethics approval was granted by the Austin Health Research Ethics Unit (10/3865) and was conducted in accordance with the Australian Code of Practice for the use of animals for scientific purposes (38).

### Dietary regime

Animals were randomly assigned to *n*-3-LC-PUFA diet condition {Specialty Feeds, Cat No. SF09-109 5% Fat High N3 Modified Rodent Diet, Glen Forest, WA, Australia [EPA 20:5 *n*-3 5% of total free fatty acid (FFA), DHA 22:6 *n*-3 23.8% of total fatty acids]}, selected as it has previously been demonstrated to reduce oxidative stress in the rat model (39) or basal diet condition (Specialty Feeds, Cat No. AIN93G; Glen Forest, WA, Australia).

### Right middle cerebral artery occlusion

Within each diet condition, animals were randomly assigned to MCAo or sham surgery condition. Surgeries were performed according to the protocol of Longa (40) and its modifications by Spratt (41). Briefly, anesthesia was induced (5% in oxygen) and maintained (2% in oxygen) with Isoflurane via a nose cone. Vital signs were monitored using iWORX (Cat No. PO2-300D, iWORX, Dover, NH, USA). Body temperature was maintained at 37°C. Atropine (0.2 ml 600 μg/ml, Pfizer Australia Pty Ltd., NSW, Australia) was administered intraperitoneally to inhibit bronchial secretions and salivation. Cerebral blood flow was measured using laser doppler flowmetry (LDF). The scalp was thinned using a dental burr and the Laser Dopler was attached to a laboratory-made rubber probe holder attached to skull with instant adhesive (Loctite 406 instant adhesive, Cat No. 265606, Henkel, Sydney, NSW, Australia). An incision was made in the neck, small branching blood vessels were cauterized, the external carotid artery was ligated, an incision made in the right external carotid artery, a 0.4-mm diameter silicone tipped suture thread inserted into the incision, passed via the right external carotid and up the internal carotid artery. MCAo was 90 min during which incisions were closed (4.0 silk sutures, Cat No. 90352, Dynek Pty Ltd., Port Adelaide, SA, Australia) and animals were removed from anesthesia. For reperfusion, animals were re-anesthetized, incisions re-opened and the MCA-occluding suture retracted. Vital signs and during reperfusion were monitored using iWORX (Cat No. PO2-300D, iWORX, Dover, NH, USA). Animal breathing was also monitored. Animals that died during the surgical or after the reperfusion procedure, or within the first 24 h after surgery, were documented and post mortem autopsy was conducted to determine cause of death. Hemorrhage was determined at the time of autopsy, by the presence of blood pooled around the base of skull. Analgesia was provided as paracetamol (Cat No. 569925, Sanofi-Aventis, Macquarie Park, NSW, Australia) crushed and dissolved into drinking water (0.15 mg/ml water). Saline was injected (i.p.) daily for 3 days following surgery [3 ml Sodium Chloride Solution (0.9%), Cat No. 7647-14-5, Tocris Bioscience, Bristol, UK]. Sham animals underwent identical procedures with the exclusion of thread insertion into the MCA. Table 1 displays animal groups, final successful numbers, and behavior testing timetable.

**Table 1:** **Experimental timeline showing order and timing of treatment and testing**.

Animal surgery and diet group	MCAo surgery and *n*-3-LC-PUFA diet	MCAo surgery and basal diet	Sham surgery and *n*-3-LC-PUFA diet	Sham surgery and basal diet
1 week prior to surgery	Diet/housing acclimation	Diet/housing acclimation	Diet/housing acclimation	Diet/housing acclimation
	Surgery	Surgery	Surgery	Surgery
Day 1–5 post-surgery	Acute motor impairment testing	Acute motor impairment testing	Acute motor impairment testing	Acute motor impairment testing
2 weeks post-surgery	Free exploration, spatial displacement recognition, and novel object exploration testing	Free exploration, spatial displacement recognition, and novel object exploration testing	Free exploration, spatial displacement recognition, and novel object exploration testing	Free exploration, spatial displacement recognition, and novel object exploration testing
2 weeks post-surgery	Behavioral testing as specified in week 2 post-surgery	Behavioral testing as specified in week 2 post-surgery	Behavioral testing as specified in week 2 post-surgery	Behavioral testing as specified in week 2 post-surgery
6 weeks post-surgery	Behavioral testing as specified in week 2 post-surgery	Behavioral testing as specified in week 2 post-surgery	Behavioral testing as specified in week 2 post-surgery	Behavioral testing as specified in week 2 post-surgery
	Sacrifice	Sacrifice	Sacrifice	Sacrifice

*MCAo, middle cerebral artery occlusion surgery condition; Sham, Sham surgery condition; Basal, basal diet fed rats; *n*-3-LC-PUFA, polyunsaturated fatty acid supplemented rats*.

### Acute stroke-related motor impairment measures

Motor impairments were assessed daily in the first 5 days post-surgery using the forelimb flexion test [MCAo affected rats hold left forelimb between 45° and 90° when lifted from base of tail (22)] the torso twisting test [MCAo affected rats show curling of the head and forelimbs toward the paralytic side of the body, when held via tail (22)] the lateral push test [MCAo affected rats show weakened resistance when pushed toward the paralytic left side (22)] and the circling [animals with infarct damage circle toward the opposite side of the damage (22)] and motor ability, observations (walking, grooming, and rearing behaviors) which collectively measure the acute inability to make co-ordinated motor movements, regularly been reported and studied in rats post MCAo compared to sham surgery (22, 23). These motor impairments are less severe in animals exposed to neuroprotective agents (24–26), thus, we aimed to determine if acute motor disability was decreased after *n*-3-LC-PUFA supplementation.

### Longer-term ischemia associated behavioral and affective disorders

The battery of behavioral tests designed to model ischemia-related affective behaviors were administered and recorded on video in the second, and then repeated in the fourth and sixth weeks post-stroke. The same animals were tested at each time point. Behavior video was scored by an observer (NC) who was blind to animal diet and surgery condition. Animal behaviors were scored by a second observer (MP), and the inter-rater reliability correlation was statistically significant, *r*^2^ = 0.97. To test for longer-term sickness behaviors and particularly depression and anxious like behaviors after surgery, we used the novel object exploration test and a modified open-field test that measures the conflict between a rat’s mutual drives to both avoid and approach unfamiliar and potentially fear inducing stimuli/environments (32, 42–47). To asses the locomotor hyperactivity often reported from as early as 5 min to 5 weeks after MCAo surgery (29, 30, 48, 49) and in rodent models of anxiety and depression (50–54), we used a modified free exploration test, which eliminates the forced exploration and related stress normally associated with the commonly used open-field test (55, 56). All behavior was recorded using a digital camcorder (Canon Legria, HG20) and was stored as a MPEG-TS video file. All apparatus were cleaned with 70% ethanol between trials (Cat No. EA043-10L, Chem-Supply, Gillman, SA, Australia).

### Free exploration test

Animals were habituated (15 min) to a laboratory constructed black Plexiglass hide box (30 cm × 15 cm × 19 cm) the day prior and re-acclimated (5 min) immediately prior to testing. The open field was a transparent Plexiglass arena (1 m^2^) illuminated (~500 lux). The hide box was positioned inside one wall of the open field. Animals were scored for locomotor hyperactivity (number of times fully emerged from the hide box, i.e., emergence number) and time spent fully emerged from hide box and moving around the arena (emergence duration) for 4 min. Given that the MCAo procedure and the associated neurodegenerative damage result in increased locomotor, activity *per se* (29, 30, 48, 49) we suggest that the above-mentioned measures may not reflect anxiety-like behaviors in MCAo operated animals, but instead may only be another measure of locomotion hyperactivity. Animals were however also scored for anxiety-like behavior that was defined as the avoidance of the center and quantified as the percentage of time spent in the center area also.

### Spatial displacement recognition

Animals were acclimated (5 min) to the testing box (40 cm × 40 cm × 60 cm) the day prior to testing. On the day of testing, animals were placed in the testing box, positioned with their nose facing the mid-point of the wall opposite the objects, to prevent an unintentional bias of placing the animal in an orientation favoring a particular object (57). Object contact was defined as when the mouth, nose, and/or paw touched the object or when the nose of the rat was within 1 cm surrounding the object. Contact judged as accidental, such as bumping the object as the animal passed or grooming behavior was not counted. The number of object contacts and the duration of these were recorded.

The spatial displacement recognition testing consisted of two habituation trials (3 min each) to two identical objects. Habituation trials were separated by a 2-min interval, during which the animal was returned to the home cage. The location of one of the two objects was then moved and the animal was placed in the testing box for a single 3 min recognition trial.

### Novel object exploration

The novel object exploration trial took place immediately after the spatial displacement recognition trial, separated only by a 2-min interval (animal returned to home cage). Testing took place in the same box used for the spatial displacement recognition test. Rats were placed in the testing box for a single 3-min trial. The familiar object was placed in one corner of the box and the novel object was placed in the opposite corner. Object placement and selection were counterbalanced across trials and animals. Contact definition is consistent with that described above for the spatial displacement recognition test.

### Tissue collection

Tissue was collected using two methods: (a) whole brain for total FFA analysis (*n* = 5 *n*-3-LC-PUFA MCAo; *n* = 6 Basal MCAo; *n* = 6 *n*-3-LC-PUFA Sham; *n* = 6 Basal Sham) animals were anesthetized via Isoflurane overdose, decapitated via guillotine, brains removed, snap frozen in liquid nitrogen (Liquid Nitrogen Services Pty Ltd., Melbourne, VIC, Australia), and stored at −80°. (b) Perfusion for infarct analysis (*n* = 6 *n*-3-LC-PUFA MCAo; *n* = 6 Basal MCAo; *n* = 6 *n*-3-LC-PUFA Sham; *n* = 6 Basal Sham). Once anesthetized, the rib cage and diaphragm were cut, a perfusion needle placed into the left ventricle of the heart and the right atrium cut. Saline (90 ml) (1.8%) was infused (5 min), followed by paraformaldehyde (PFA) (4%) (270 ml) (15 min), using a perfusion pump (Peri-Star Pro 4-channel, high rate pump, Cat No. PERIPRO-4HS, World Precision Instruments, Hilton, SA, Australia) (18 ml/min). Brains were removed and placed directly into PFA (4%) for 24 h, before being changed to 30% sucrose, where they remained until the time of paraffin embedding.

### Fatty acid analysis

To confirm that the *n*-3-LC-PUFA supplementation resulted in changes in brain phospholipids levels of fatty acids, whole brain frozen tissue was ground up, and tissue lipids extracted by dichloromethane/methanol (2:1) overnight, as described by Sinclair et al. (58). Samples were filtered, saline added (1 ml of 0.9%), and vortexed (1 min). Samples were centrifuged (1500 × *g*, 10 min) to separate the aqueous and organic phases at room temperature. The organic phase containing the lipid was removed and transferred to a new glass tube and evaporated under a stream of nitrogen. The lipid extract was reconstituted in 200 μl of dichloromethane and lipids were then separated by thin layer chromatography (TLC). The lipid extracts were spotted onto silica gel plates (silica gel 60 G, Merck, Germany) and developed in 85:15:2 (v/v) petroleum ether: diethyl ether: acetic acid in paper-lined tanks. The lipids were visualized with 0.1% (w/v) 2′,7′-dichlorofluorescein indicator in ethanol (Scharlau, Spain). The phospholipid bands from the samples were scraped off into glass screw-capped tubes and were reacted with 5% H_2_SO_4_ in 100% methanol (3 h at 80°C) to form the fatty acid methyl esters (FAMEs). FAME were isolated (100% petroleum ether) and stored in glass (−20°C). Purified FAME were isolated and identified using an Agilent Technologies 7890A GC System (Agilent Technologies, Santa Clara, CA, USA) equipped with an Omegawax 250 capillary column (30 m × 0.25 mm internal diameter, 0.25 μm film thickness, Supelco, Bellefonte, PA, USA), a flame ionization detector (FID), an Agilent Technologies 7693 auto sampler, and a split injection system (split ratio 50:1). The injection volume was 1 μl, the injector and detector temperature were 300 and 270°C, respectively. The temperature program was 50–190 at 20°C min^−1^, then from 190 to 250 at 4°C min^−1^, and held at 250°C for 8 min. The carrier gas was helium at 1.18 ml min^−1^, at a constant flow. Each of the fatty acids was identified relative to known external standards (a series of mix and individual standards from Sigma-Aldrich, Inc., St. Louis, MO, USA and from Nu-Chek Prep Inc., Elysian, MN, USA). The resulting peaks were then corrected by the theoretical relative FID response factors (59) and quantified relative to the internal standard.

### Infarct volume analysis and area of damage

Brains were cut (2 mm coronal sections) using an acrylic rat brain matrix. Tissue was processed using a closed linear Tissue Processing System (Cat No. TPC 15, MEDITE GmbH, Wollenweberstr, Burgdorf, Germany). Tissue was embedded in molten paraffin wax (Cat No. Leica EG1150 H, Leica-microsystems, Ernst-Leitz-Straße, Wetzlar, Germany), and stored at room temperature. Tissue was cut at room temperature (7 μm) using a rotary microtome (Cat No. Leica 2040, Leica-microsystems, Ernst-Leitz-Straße, Wetzlar, Germany) suspended at 40°C in a tissue flotation bath and attached to silane coated slides (Cat No. CS2460100MK, Micro-glass, Grale Scientific, Melbourne, VIC, Australia). Tissue slides were dried at 49°C, incubated at 32°C for 24 h, and stained with Hematoxylin (5 g Hematoxylin; Cat No. 340374T, VWR International Pty Ltd., Murarrie, QLD, Australia) and Eosin (10 g Eosin Y; Cat No. E-4382, Sigma-Aldrich, St. Louis, MO, USA). Slides were de-waxed in Histosol (Cat No. CP L HISTOSOL 08, HD Scientific, Wetherill Park, NSW, Australia), washed in ethanol, rinsed in dH_2_O, dehydrated in ethanol, followed by two Histosol washes. Slides were rehydrated in ethanol rinsed in dH_2_O and stained with filtered Harris Hematoxylin. Slides were rinsed in dH_2_O, stained in filtered Eosin Y, washed in dH_2_O, and dehydrated in ethanol. This was followed histosol washes and cover slipping (DPX, Cat No. 1019790500, Merck KGaA, Darmstadt, Germany). Stained tissue was examined and using bright field microscopy (Nikon Eclipse 80i, Nikon Instruments Europe) with a Nikon DIGITAL SIGHT DS-U1 camera. Photographs were stored as JPEG Image files. Areas of tissue damage were analyzed using Stereo Investigator Version 6 software.

### Data analysis

The present study uses both parametric and non-parametric data analysis techniques as appropriate. This study consists of four independent groups [2 × 2 (*n*-3-LC-PUFA MCAo; Basal MCAo; *n*-3-LC-PUFA sham; Basal Sham)] and has both single time point and repeated measures outcomes. Where data met the assumption of normality, appropriate ANOVAs, as described below are used. All measures of longer-term behavioral outcomes however did not meet the assumption of normality and were unable to be transformed (except spatial displacement recognition) and thus non-parametric data analyses techniques have been used. It is important to note however, that IBM SPSS Statistics does not offer a non-parametric equivalent to the mixed design ANOVA (the appropriate parametric analysis technique to analyze the longer-term behavioral outcome data). The non-parametric Friedman’s ANOVA allows a comparison of two or more related groups, but is not appropriate for the current study, where groups are independent. The Kruskal–Wallis is appropriate for the current data, as it allows one to compare differences between two or more independent groups (i.e., *n*-3-LC-PUFA MCAo vs. Basal MCAo/*n*-3-LC-PUFA sham vs. Basal Sham) (60). To determine where group differences exist, Mann–Whitney *post hoc* tests were used. In order to control for type 1 errors, only the most important/relevant *post hoc* Mann–Whitney comparisons were made, thus differences between diet groups; within each of the different surgery conditions, at weeks 2, 4, and 6 post-surgery, have not been assessed. We determined that the most important/relevant *post hoc* Mann–Whitney comparisons are: (a) differences between MCAo operated and Sham operated rats and (b) differences between *n*-3-LC-PUFA and basal diet fed rats, at weeks 2, 4, and 6 post-surgery, and thus these group differences have been assessed.

For single time point outcome measures, where the assumptions of normality were met [infarct volume and total fatty acid levels (micrograms) in brain phospholipids] ANOVAs were conducted. For outcome variables measured at multiple time points [body weight, food, and water consumption (grams), acute stroke-related motor impairment and spatial displacement recognition] mixed design ANOVAs were conducted (effect of diet condition within surgery condition measured at multiple time points). LSD *post hoc* test with a Bonferroni correction were used. Normality of data from behavioral tests was assessed using Q-Q plots and histograms. Outliers were screened for using box plots and no data was deleted, the assumptions of homogeneity was checked using Levene’s Test of Equality of Variance, and sphericity was checked using Mauchly’s Test of Sphericity. A square root transformation was conducted on spatial displacement recognition data to achieve a normal distribution. Spearman correlations were used to detect correlations between emergence behavior and infarct damage. Spearman correlations were also conducted between both the number of emergences from the hide box into the open field and the duration of time spend moving around the open-field arena, and the percentage of emergence time spent in the center of the open-field arena. Independent sample *t* tests were used to compare mean differences in animal weight and the number of animals that died from hemorrhage following the reperfusion component of the surgery, as reported below, between diet groups at time of surgery.

## Results

### Using parametric data analysis techniques

#### Increased risk of reperfusion related hemorrhage following reperfusion among *n*-3-LC-PUFA supplemented animals

**Figure 1:**
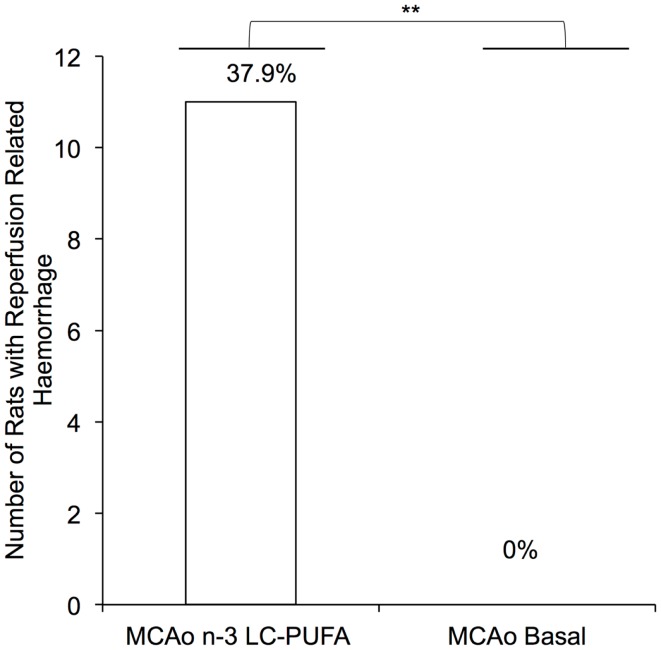
**Number of MCAo operated rats that experienced reperfusion related hemorrhage, between diet conditions**. MCAo, middle cerebral artery occlusion surgery condition; Basal, basal diet fed rats; *n*-3-LC-PUFA, polyunsaturated fatty acid supplemented rats; *n* = 11 *n*-3-LC-PUFA MCAo; *n* = 0 basal MCAo.

An unexpected finding of the present research that has not previously been reported was that 39% (*N* = 11) of all the *n*-3-LC-PUFA diet acclimated animals that underwent MCAo surgery experienced subarachnoid hemorrhagic bleeding following the reperfusion component of the surgery, as illustrated in Figure 1. Upon unblinding of groups, we found that this surgical complication was only seen in the *n*-3-LC-PUFA diet acclimated animals and did not occur in any of the basal diet fed rats. We did note any intracerebral bleeding in animals in either of the diet groups. Independent sample *t* tests indicate that the difference in the rate of reperfusion related bleeding between diet groups was significant, *t*(17) = 3.29, *p* < 0.01. These animals were obviously excluded from the final cohort numbers. A further 16 rats who did not experience hemorrhagic bleeding were culled after surgery, due to MCAo symptoms too large for the animal to survive. All these rats were replaced and thus the total number of animals used in the present study was 75 (*n* = 29 *n*-3-LC-PUFA MCAo; *n* = 22 Basal MCAo; *n* = 12 *n*-3-LC-PUFA sham; *n* = 12 Basal Sham). Unfortunately, as the death of the *n*-3-LC-PUFA fed animals following the reperfusion component of the surgery was unexpected we were unable to prepare the tissue for further experimental analysis.

### Animal weight from time of surgery until sacrifice

Surviving animal (*N* = 48) mean bodyweight was 337 (SD = 36.5) grams at time of surgery. There was no main effect for diet condition in mean bodyweight at time of surgery, *F*(1, 47) = 1.14, *p* = 0.29 (*n*-3-LC-PUFA *M* = 332, SE = 6.7, Basal *M* = 342, SE = 6.4). Between surgery conditions, sham operated rats were slightly lighter than MCAo operated animals, *F*(1, 47) = 14.24, *p* < 0.01 (MCAo *M* = 354, SE = 6.6, Sham *M* = 320, SE = 6.6). There was no interaction between diet and surgery condition on animal body weight at the time of surgery.

### Food and water consumption from time of surgery until sacrifice

**Figure 2:**
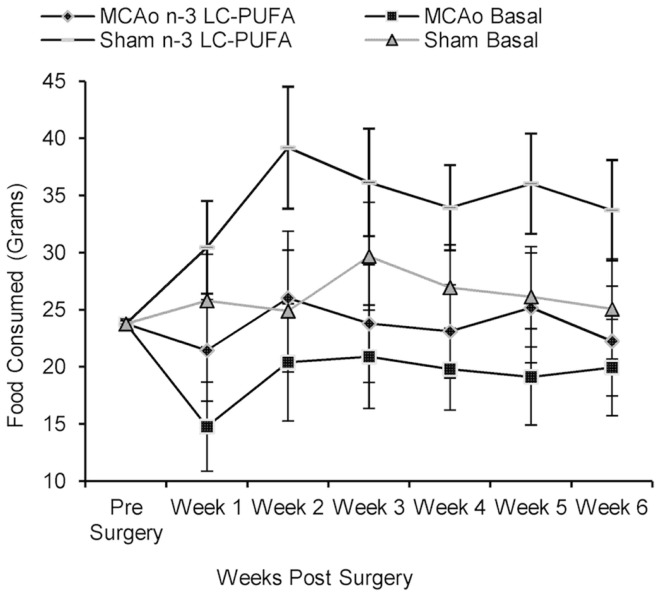
**Mean food consumption (shown with confidence intervals), between stroke and diet conditions from week 1 until week 6 post-surgery**. MCAo, middle cerebral artery occlusion surgery condition; Sham, Sham surgery condition; Basal, basal diet fed rats; *n*-3-LC-PUFA, polyunsaturated fatty acid supplemented rats; week 1, week one post-surgery; week 2, week two post-surgery; week 3, week three post-surgery; week 4, week four post-surgery; week 5, week five post-surgery; week 6, week six post-surgery. *n* = 11 *n*-3-LC-PUFA MCAo; *n* = 13 Basal MCAo; *n* = 12 *n*-3-LC-PUFA sham; *n* = 12 Basal Sham.

**Figure 3:**
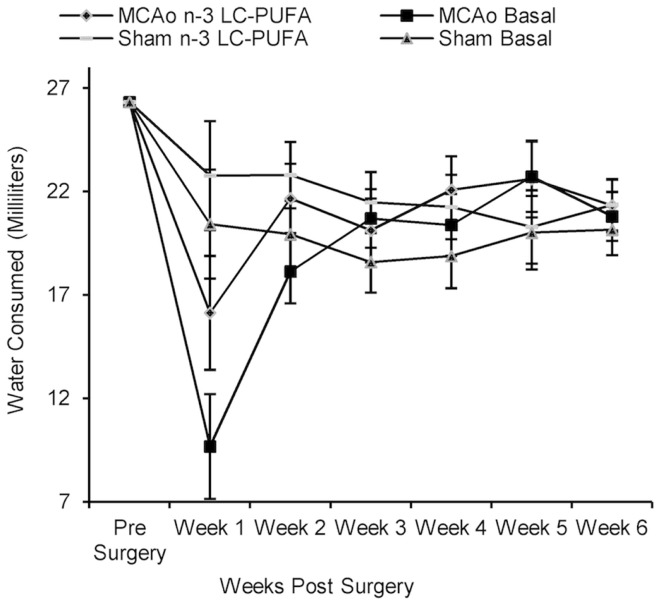
**Mean water consumption (shown with confidence intervals), between surgery and diet conditions from week 1 until week 6 post-surgery**. Note the large drop in water consumption immediately after surgery and the rapid recovery in drinking behavior, by week 2 post-surgery, and by the time that ischemia associated motor impairments were no longer obvious. MCAo, middle cerebral artery occlusion surgery condition; Sham, Sham surgery condition; Basal, basal diet fed rats; *n*-3-LC-PUFA, polyunsaturated fatty acid supplemented rats; week 1, week one post-surgery; week 2, week two post-surgery; week 3, week three post-surgery; week 4, week four post-surgery; week 5, week five post-surgery; week 6, week six post-surgery. *n* = 11 *n*-3-LC-PUFA MCAo; *n* = 13 Basal MCAo; *n* = 12 *n*-3-LC-PUFA sham; *n* = 12 Basal Sham.

Rats did not differ in food and water consumption at the time of surgery (see Figures 2 and 3). On average at the time of surgery, sham operated *n*-3-LC-PUFA diet fed animals were consuming ~206 mg of EPA, and 909 mg of DHA per day, per kilogram of total body weight. MCAo operated *n*-3-LC-PUFA diet fed rats were consuming 181 mg of EPA and 800 mg of DHA per day, per kilogram of total body weight.

All animals increased in bodyweight from week 1 until week 6 post-surgery, *F*(59, 205) = 30.43, *p* < 0.01. Food, *F*(5, 215) = 5.79, *p* < 0.01 and water, *F*(5, 220) = 13.10, *p* < 0.01 consumption increased between week 1 and week 6 post-surgery. A main effect of surgery condition was seen and as MCAo operated animals consumed less food from week 1 until week 6 post-surgery, *F*(1, 43) = 25.46, *p* < 0.01 and drank less water, *F*(1, 44) = 4.40, *p* < 0.05, than sham operated animals. A main effect of diet condition was also seen over the same period of time, as *n*-3-LC-PUFA supplemented animals consumed more food, *F*(1, 43) = 12.47, *p* < 0.01, and water, *F*(1, 44) = 18.06, *p* < 0.01 than basal diet fed rats. No interaction effects were seen.

During the 6-weeks post-surgery, sham operated *n*-3-LC-PUFA diet fed animals consumed an average of 272 mg of EPA and 1200 mg of DHA daily, per kilogram of total body weight. On average, MCAo operated *n*-3-LC-PUFA diet fed animals consumed 189 mg of EPA and 833 mg of DHA daily, per kilogram of total body weight. Figures 2 and 3 show the mean food consumption in grams and mean water consumption in milliliters respectively, between stroke and diet conditions, between week 1 and 6 post-surgery.

### Fatty acid analysis

**Figure 4:**
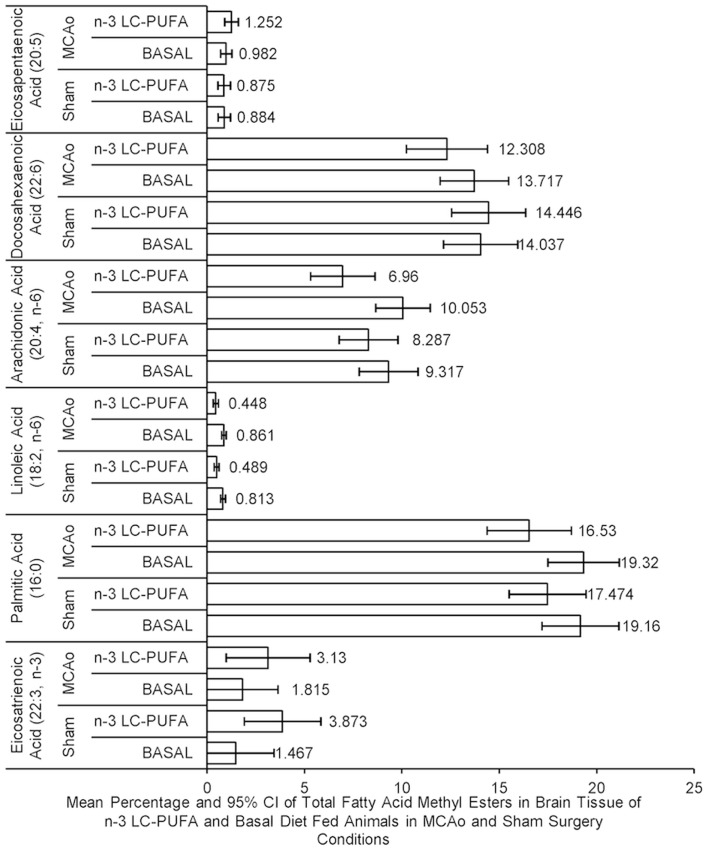
**Mean percentage and confidence intervals of whole brain phospholipid fatty acids in fatty acid methyl esters between diet and surgery conditions**. Note that although no difference between dietary conditions is seen in the level of eicosapentaenoic acid (20:5) or docosahexaenoic acid (22:6), the levels of fatty acids (palmitic acid [16:0], arachidonic acid [20:4], and linoleic acid [18.2]) were higher in basal diet fed animals, compared to *n*-3-LC-PUFA fed animals, in whole brain tissue. By comparison, *n*-3-LC-PUFA fed animals are shown to express more omega-3 derived fatty acids [eicosatrienoic acid (22:3)] than basal diet fed animals. No differences are seen in fatty acid methyl esters between surgery groups. −20:5 *n*-3, eicosapentaenoic acid; 22:6 *n*-3, docosahexaenoic acid; 20:4 *n*-6, arachidonic acid; 18:2 *n*-6, linoleic acid; 16:0, palmitic acid; 22:3 *n*-3, eicosatrienoic acid. MCAo, middle cerebral artery occlusion surgery condition; Sham, Sham surgery condition; Basal, basal diet condition; *n*-3-LC-PUFA, *n*-3 L-C-PUFA enriched diet condition. *Denotes statistically significant at *p* = 0.05, **denotes statistically significant at *p* = 0.01. *n* = 11 *n*-3-LC-PUFA MCAo; *n* = 13 Basal MCAo; *n* = 12 *n*-3-LC-PUFA sham; *n* = 12 Basal Sham.

Fatty acid levels are reported as percentage of total FAME. There were no main effects of surgery condition on fatty acid levels. ANOVA showed a main effect for diet condition of fatty acid levels, as *n*-3-LC-PUFA fed animals showed less *n-*6 in whole brain tissue than animals fed the basal diet, evidenced by less arachidonic acid (AA; 20:4, *n*-6), *F*(1, 23) = 8.01, *p* < 0.05, linoleic acid (LA; 18:2, *n*-6), *F*(1, 23) = 37.41, *p* < 0.01 and palmitic acid (16:0) in brain phospholipids, *F*(1, 23) = 5.51, *p* < 0.05. A trend for higher levels of eicosatrienoic acid (ETA; 22:3, *n*-3), *F*(1, 23) = 3.85, *p* = 0.06, among *n*-3-LC-PUFA fed rats than basal fed rats was seen. No main significant main effect of diet was seen in the amount of DHA (22:6, *n-*3), *F*(1, 23) = 0.30, *p* > 0.05, or EPA (20:5, *n*-3), *F*(1, 23) = 0.72, *p* > 0.05. There were no significant interaction effects. Figure 4 illustrates differences in the mean fatty acid levels in the brain phospholipids between diet and surgery conditions.

### Infarct volume analysis and area of damage

**Figure 5:**
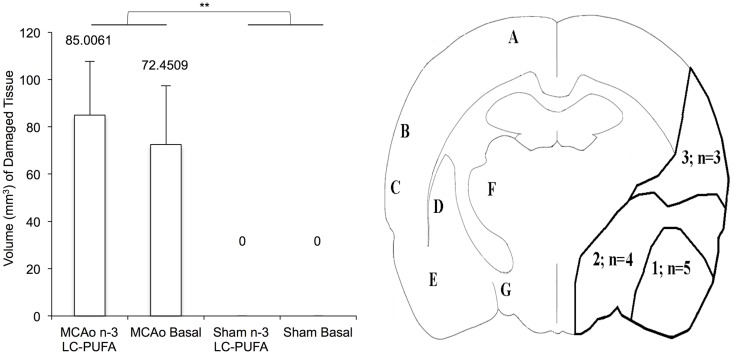
**Mean volume (cubic millimeter) of damaged tissue (shown with standard error), between stroke and diet conditions at week 6 post-surgery and outline of infarct area 6-weeks after middle cerebral artery occlusion**. Variability was seen in infarct size and animals (*n* = 5) with smaller infarcts generally showed atrophy in the area 1 outline, animals with larger infarcts (*n* = 5) showed atrophy in the both area 1 and 2. (1) area of atrophy in animals with smaller infarcts; (2) area of atrophy additional to area A in animals with larger infarcts; A, motor areas M1 and M2; B, somatosensory cortex; C, auditory cortex; D, caudate putamen; E, amygdala region; F, ventral posterolateral thalamic nucleus/ventral posteromedial thalamic nucleus; G, lateral hypothalamic area. *Denotes statistically significant at *p* = 0.05, **denotes statistically significant at *p* = 0.01. *n* = 11 *n*-3-LC-PUFA MCAo; *n* = 13 Basal MCAo; *n* = 12 *n*-3-LC-PUFA sham; *n* = 12 Basal Sham.

Mean volume (cubic millimeter) of damaged tissue between stroke and diet conditions at week 6 post-surgery and an outline of infarct area 6-weeks after MCAo, is illustrated in Figure 5. MCAo affected animals showed a greater volume (cubic millimeter) of damaged tissue, *F*(3, 20) = 21.82, *p* < 0.01 (*M* = 78.72, SE = 16.12) when compared to sham operated animals that did not show any infarct damage (*M* = 0.0, SE = 0.0). No main effect on infarct volume was found for diet condition, *F*(3, 20) = 0.14, *p* > 0.05 (*n*-3-LC-PUFA, *M* = 36.23, SE = 16.15; basal, *M* = 42.50, SE = 16.76). No significant interaction effects were seen. In MCAo operated animals, right hemisphere infarct was seen in cortical and subcortical tissue. Variability was seen in infarct size. The area labeled A in Figure 5 outlines the infarct region typically seen in animals with smaller infarcts [*n* = 5 (*n*-3-LC-PUFA diet = 2)]. Both the areas labeled A and B outline the infarct region seen in animals with medium infarcts [*n* = 4 (*n*-3-LC-PUFA diet = 1)]. The areas outlined A, B, and C outlines the infarct region seen in animals with larger infarcts [*n* = 3 (*n*-3-LC-PUFA diet = 3)].

### Acute stroke-related motor impairment measures

Main effects for surgery condition were found for all motor impairment measures, with MCAo operated animals showing greater impairment, forelimb flexion, *F*(1, 36) = 19.32, *p* < 0.01, torso twisting, *F*(1, 35) = 40.29, *p* < 0.01, lateral push, *F*(1, 36) = 36.34, *p* < 0.01, circling, *F*(1, 35) = 52.52, *p* < 0.01 and motor ability, *F*(1, 36) = 23.04, *p* < 0.01. A significant main effect of diet condition was seen for lateral push, *F*(1, 36) = 4.34, *p* < 0.05.

Interaction effects between diet and surgery condition were seen for lateral push, *F*(1, 36) = 4.34, *p* < 0.05, and motor ability *F*(1, 36) = 5.22, *p* < 0.05, with *n*-3-LC-PUFA MCAo operated rats showing a smaller impairment than basal fed rats (lateral push: MCAo Basal *M* = 0.20, MCAo *n*-3-LC-PUFA *M* = 0.10; motor ability: MCAo Basal *M* = 0.40, MCAo *n*-3-LC-PUFA *M* = 0.20) and sham exposed rats showing minimal impairment, irrespective of diet condition (lateral push: Sham Basal *M* = 0.0, Sham *n*-3-LC-PUFA *M* = 0.0; motor ability: Sham Basal *M* = 0.0, Sham *n*-3-LC-PUFA *M* = 0.05).

Repeated measures ANOVA showed a decrease in motor impairment from day 1, until day 5 post-surgery. A statistically significant main effect for days after surgery was seen in the torso twisting test, *F*(3, 118) = 3.23, *p* < 0.05, lateral push test, *F*(4, 144) = 3.01, *p* < 0.05, circling, *F*(4, 140) = 4.78, *p* < 0.01, and motor ability observations, *F*(4, 144) = 5.65, *p* < 0.01.

### Longer-term ischemia associated behavioral disorders

#### Spatial displacement recognition – classically considered a test for spatial memory deficits

No main effects were found for surgery condition, *F*(1, 44) = 0.14, *p* = 0.71, or diet condition, *F*(1, 44) = 0.09, *p* = 0.77 at any testing time point post-surgery. There were no interaction effects. All animals were found to explore the displaced object equally.

### Non-parametric data analysis techniques

#### Free exploration test – locomotor hyperactivity

Measures of locomotor hyperactivity were significantly correlated with each other (rho = 0.82, *p* < 0.00), indicating that these two outcomes are likely reflecting the same construct. Spearman correlation showed a positive correlation between total infarct volume and emergence number at weeks 2 (rho = 0.47, *p* < 0.05) and 6 (rho = 0.44, *p* < 0.05) and a trend at week 4 (rho = 0.39, *p* = 0.06).

**Figure 6:**
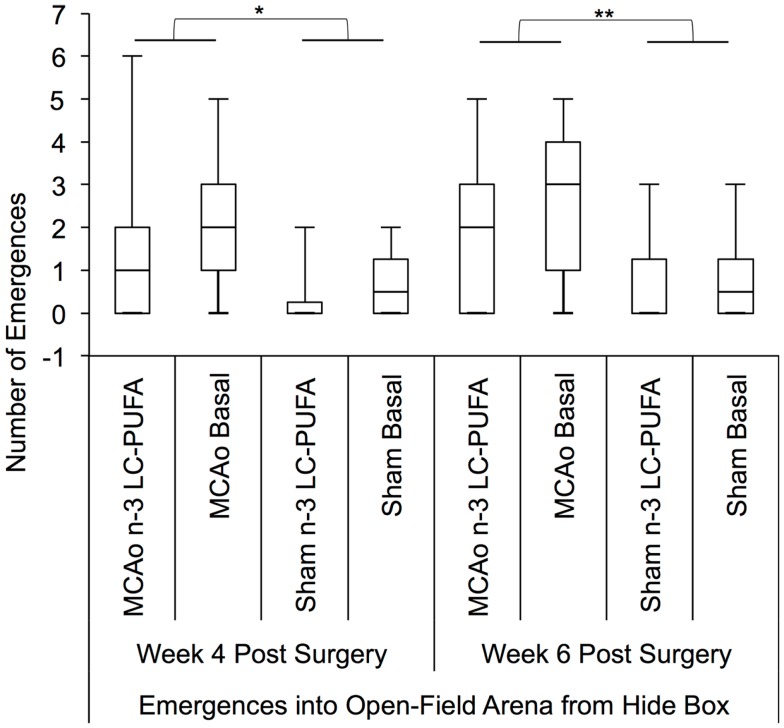
**Box plots showing significant *post hoc* differences in number of emergences into open field, between diet and surgery conditions**. Visual inspection of this Figure indicates that sham surgery-operated animals were less active than MCAo operated animals and that in each surgery condition, at each time point, the *n*-3-LC-PUFA animals emerged from the hide box less often, suggesting less hyperactivity. MCAo, middle cerebral artery occlusion surgery condition; Sham, Sham surgery condition; Basal, basal diet fed rats; *n*-3-LC-PUFA, polyunsaturated fatty acid supplemented rats. *n* = 11 *n*-3-LC-PUFA MCAo; *n* = 13 Basal MCAo; *n* = 12 *n*-3-LC-PUFA sham; *n* = 12 Basal Sham.

**Figure 7:**
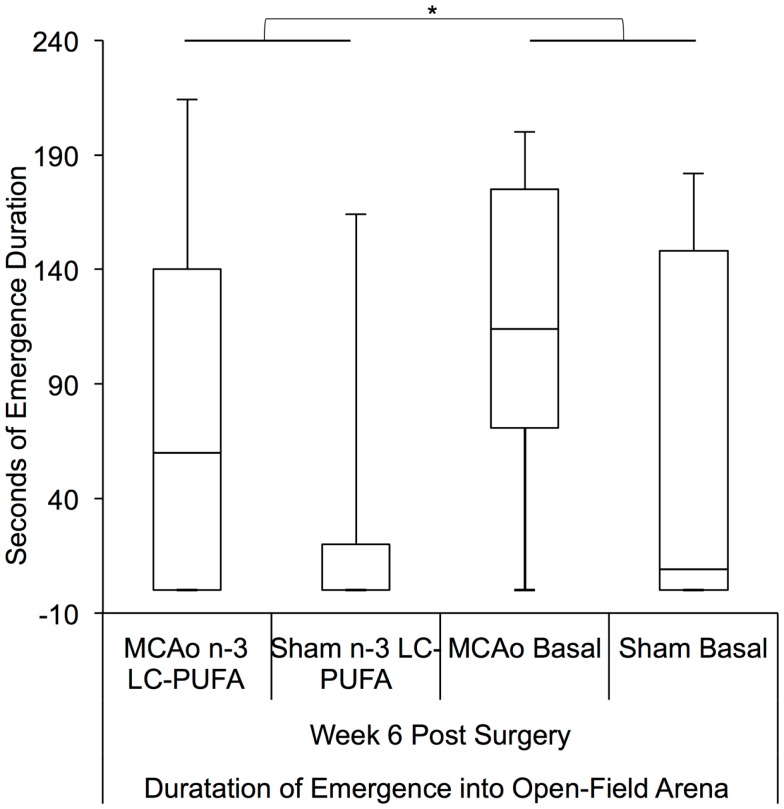
**Box plots showing significant *post hoc* differences in emergence duration between diet and surgery conditions at 6 weeks post-surgery**. Visual inspection of this Figure indicates that sham surgery-operated animals spent less time moving around in the open-field arena compared to MCAo operated animals. Furthermore, in each surgery condition, *n*-3-LC-PUFA diet fed animals spent less time moving around the open-field arena than do basal diet fed animals. MCAo, middle cerebral artery occlusion surgery condition; Sham, Sham surgery condition; Basal, basal diet fed rats; *n*-3-LC-PUFA, polyunsaturated fatty acid supplemented rats. *n* = 11 *n*-3-LC-PUFA MCAo; *n* = 13 Basal MCAo; *n* = 12 *n*-3-LC-PUFA sham; *n* = 12 Basal Sham.

Kruskal–Wallis non-parametric analysis showed that diet and surgery groups differed in the number of full body emergences from the hide box into the open field, at weeks 4 [*H*(3) = 7.81, *p* = 0.05] and 6 [*H*(3) = 10.34, *p* < 0.05], and in the total duration spent emerged and moving in the open field at week 6 [*H*(3) = 10.00, *p* < 0.05]. *Post hoc* Mann–Whitney tests show that MCAo affected animals made more emergences from the hide box into the open field than sham affected animals at weeks 4, *U* = 179.5, *z* = −2.39, *p* < 0.05 (MCAo, mdn = 1, IQR = 2.75; Sham, mdn = 0; IQR = 1) and 6, *U* = 155, *z* = −2.87, *p* < 0.01 (MCAo, mdn = 2, IQR = 3.5; Sham, mdn = 0; IQR = 1.75). Emergence duration similarly differed between surgery groups at week 6, *U* = 1.72, *z* = −2.48, *p* < 0.05 with MCAo animals (mdn = 145; IQR = 177) spending more seconds emerged than sham (mdn = 8; IQR = 116) affected animals, indicative of more stroke-related locomotor hyperactivity. A comparison of diet conditions showed that *n*-3-LC-PUFA animals (mdn = 15; IQR = 145) spent fewer seconds out in the open-field area than basal fed rats (mdn = 95; IQR = 189) at week 6 post-surgery, *U* = 195, *z* = −1.99, *p* < 0.05, suggesting that *n*-3-LC-PUFA supplementation reduced locomotor hyperactivity by 6 weeks post-surgery. Figures 6 and 7 depict box plots showing significant *post hoc* differences between groups.

### Free exploration test – classically a test of anxiety-like behavior

The above outlined measures of locomotor activity were both found to be negatively correlated with the percentage of total emergence time spent in the center of the open-field arena [emergence number (rho = −0.35, *p* < 0.01) and duration of time spent moving in the open-field arena (rho = −0.45, *p* < 0.01)], indicating that they are unlikely to reflect anxiety-like behavior in operated animals.

**Figure 8:**
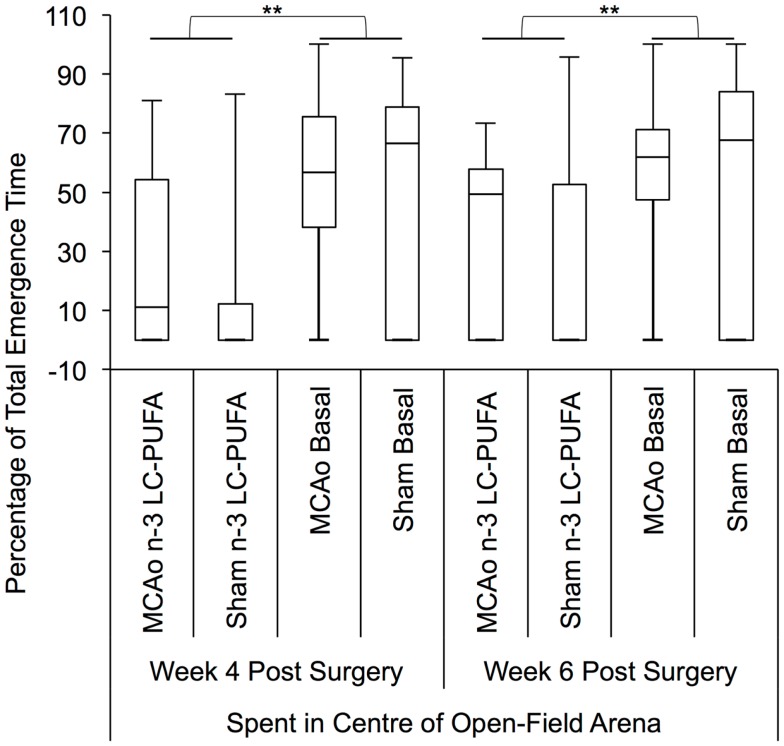
**Box plots showing significant *post hoc* differences in percentage of total emergence time spent in center of open field, compared to the side wall of the open-field arena, between diet and surgery conditions**. Visual inspection of this figure indicates that *n*-3-LC-PUFA diet fed animals spent more time in the center of the arena, than did basal diet fed animals. No effects of surgery condition on percentage of time spent in the center of the arena can be seen. MCAo, middle cerebral artery occlusion surgery condition; Sham, Sham surgery condition; Basal, basal diet fed rats; *n*-3-LC-PUFA, polyunsaturated fatty acid supplemented rats. *n* = 11 *n*-3-LC-PUFA MCAo; *n* = 13 Basal MCAo; *n* = 12 *n*-3-LC-PUFA sham; *n* = 12 Basal Sham.

Non-parametric Kruskal–Wallis test indicated that surgery and diet groups differed in the percentage of total emergence time spent in the center of the open-field arena at 4 weeks post-surgery, as compared to side wall of the arena, *H*(3) = 7.79, *p* < 0.05. At 6 weeks post-surgery, an effect for a difference in the percentage of total emergence time spent in the center of the open field arena approached significance, *H*(3) = 7.25, *p* = 0.06. The appropriate *post hoc* Mann–Whitney test indicated that rats supplemented with *n*-3-LC-PUFA spent significantly a greater percentage of total emergence time in the center of the arena at week 4, *U* = 16.2.0, *z* = −2.72, *p* < 0.01 (*n*-3-LC-PUFA, mdn = 66, IQR = 80; Basal, mdn = 0; IQR = 51), and week 6, *U* = 161.5, *z* = −2.65, *p* < 0.01 (*n*-3-LC-PUFA, mdn = 70, IQR = 40; Basal, mdn = 40; IQR = 56), compared to basal diet fed rats. *Post hoc* tests showed no differences between surgery conditions. Figure 8 depicts box plots showing significant *post hoc* differences between groups.

### Novel object exploration – classically considered a test of approach avoidance behaviors

**Figure 9:**
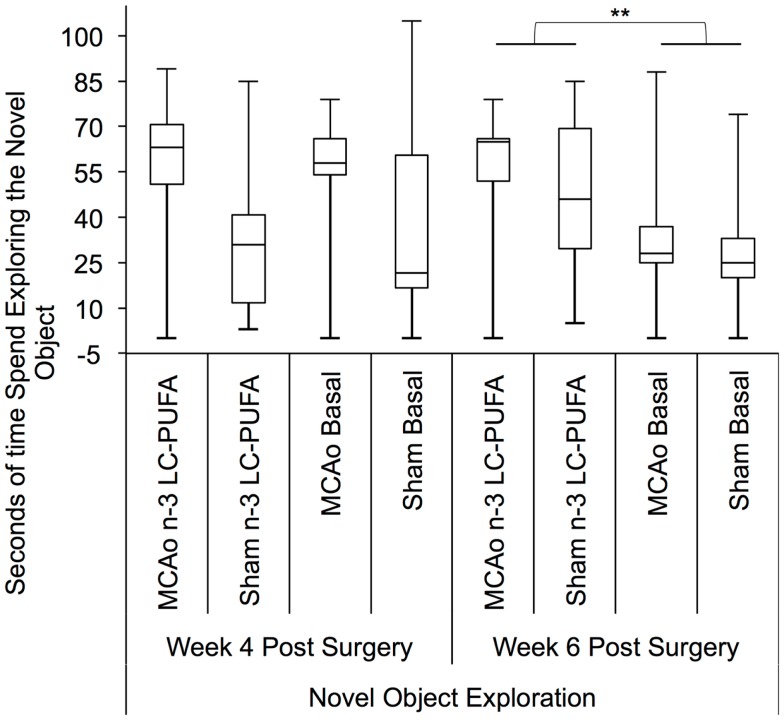
**Box plots showing significant *post hoc* differences in time spent exploring novel object exploration between diet and surgery conditions both at 4 and 6 weeks post-surgery**. Visual inspection of this figure indicates that at both time points sham surgery-operated animals supplemented with *n*-3-LC-PUFA diet spent more time exploring the novel object than sham operated basal diet fed animals. MCAo surgery-operated animals supplemented with *n*-3-LC-PUFA diet appear to have spent slightly more time exploring the novel object than MCAo operated basal diet fed animals. MCAo, middle cerebral artery occlusion surgery condition; Sham, Sham surgery condition; Basal, basal diet fed rats; *n*-3-LC-PUFA, polyunsaturated fatty acid supplemented rats. *n* = 11 *n*-3-LC-PUFA MCAo; *n* = 13 Basal MCAo; *n* = 12 *n*-3-LC-PUFA sham; *n* = 12 Basal Sham.

Approach–avoidance conflict was studied using the novel object exploration test. Box plots are presented in Figure 9. Surgery and diet groups differed in novel object exploration time at 4 weeks, *H*(3) = 9.00, *p* < 0.01, and 6 weeks post-surgery, *H*(3) = 12.50, *p* < 0.01.

*Post hoc* comparisons between surgery conditions showed MCAo affected animals explored the novel object less (in seconds) than sham operated animals at week 4 post-surgery, *U* = 175, *z* = −2.32, *p* < 0.05 (MCAo, mdn = 6, IQR = 17; Sham, mdn = 17; IQR = 42). Animals did not demonstrate any MACo related motor deficits that might have been responsible for changes in exploration behaviors. *Post hoc* comparison of diet conditions showed that the *n*-3-LC-PUFA supplemented animals in each surgery condition spent more significantly more seconds exploring the novel object than the basal fed animals at week 6, *U* = 144, *z* = −2.95, *p* < 0.01 (*n*-3-LC-PUFA, mdn = 31, IQR = 23; Basal, mdn = 14; IQR = 15). At weeks 4, an effect for greater exploration of the novel object by *n*-3-LC-PUFA supplemented animals compared to basal diet fed animals approached significance, *U* = 198, *z* = −1.85, *p* = 0.06 (*n*-3-LC-PUFA, mdn = 17, IQR = 28; Basal, mdn = 5; IQR = 18).

## Discussion

We aimed to investigate effects of on-going *n*-3-LC-PUFA supplementation on surgery induced sickness behaviors, motor impairment, and anxiety-like and locomotor behaviors. To our knowledge, this is the first rodent study to explore the effects of *n*-3-LC-PUFA supplementation against persistent anxiety-like and locomotor behaviors post-surgery.

Surprisingly, increased reperfusion related hemorrhagic risk in MCAo *n*-3-LC-PUFA supplemented rats occurred, even after only a short acclimation period (1 week prior to surgery). Thus, while *n*-3-LC-PUFA supplementation may reduce affective and behavioral disorders, it also appears to be associated with increased risk of hemorrhagic bleeding. Unfortunately, the death of the *n*-3-LC-PUFA fed animals following the reperfusion component of the surgery was unexpected, we were unable to rescue the tissue for further experimental analysis, and thus, this preliminary data should be interpreted with caution.

As far as we are aware, *n*-3-LC-PUFA related hemorrhagic bleeding has not previously been reported in the context of experimental MCAo. Our findings are consistent however with previous rodent studies, using an experimental model of intracerebral hemorrhage. One study demonstrated that 41% of animals died due to excessive bleeding after experimentally induced intracerebral hemorrhage, when fed a diet supplemented with 1% EPA/DHA, for a period of 5 weeks prior to surgery. The authors concluded that the increased mortality might be due to DHA and EPA associated increases in oxidative damage (18). Indeed, FFA expression is seen to increase at 7 days after experimentally induced hemorrhagic stroke in rodents, with expression returning to baseline levels at 14 days post-surgery (61). Another study indicated that 2 weeks of supplementation with fish oil (9% DHA and 12.9% EPA) prior to intracerebral hemorrhage, is associated with increased cerebral blood flow in the peri-infarct region and contralateral hemisphere, at 6 h after surgery, in male Sprague-Dawley rats. Animals fed the fish oil diet also showed greater disability in fine motor control, compared to animals fed a sunflower oil enriched diet (rich in omega-6). The above-mentioned disabilities were present in spite of no statistically significant difference in infarct size, between animals in different diet groups (62). A second study indicated high omega-6 AA was negatively associated with gastric hemorrhage, and that 7 days of fish oil supplementation decreases AA in liver phosphatidylcholine. Those authors speculated that fish oil may increase gastric bleeding due to modulating the permeability of cell membranes in the gastric mucosa (63) in their inbred rats that were fed a 7.5% fish oil diet. Given the limited research, we are unable to specify if hooded-Wistar rats differ in bleeding times, rates of platelet aggregation, and responsiveness to dietary EPA/DHA supplementation compared to other rat strains. After subarachnoid hemorrhage, both animal and clinical studies demonstrate that EPA reduces vasospasm, or contraction of smooth muscle cells, in part via inhibition of TXA_2_, a thromboxane that increases platelet aggregation. Conversely, the F_2_-isoprostanes, derived from the omega-6 fatty AA, are associated with increased risk of subarachnoid hemorrhage related vasospasm (64–69).

In Greenland Eskimos, high PUFA consumption has long been associated with increased incidence of hemorrhagic stroke (70, 71). In clinical populations, a small study involving 20 individuals, demonstrated that PUFAs expression is elevated in the cerebral spinal fluid (CSF) of individuals who experienced hemorrhagic stroke, at 24 until 48 h after insult, when compared to non-stroke affected individuals (67). A later clinical study by the same authors, involving 25 patients, who experienced either hemorrhagic or ischemic stroke, similarly showed elevated PUFA expression in CSF, within 48 h following insult, compared to healthy individuals (72). Additionally, PUFA expression was positively correlated with worse outcome at the time of hospital discharge. The authors interpreted these data to indicate the PUFA expression may be a marker of stroke outcome. These authors however found no difference in PUFA expression between individuals who experienced a hemorrhagic stroke compared to those who experienced ischemic stroke (72). Animal studies have also documented elevated PUFA expression after ischemic stroke (73–75) and thus the role of PUFAs in hemorrhagic stroke specifically is still unclear.

Stroke survivors are already considered particularly high risk for hemorrhagic transformation (76–79). Therefore, some authors argue that it is prudent to ensure that patients who are classified as high risk for hemorrhage discontinue *n*-3-LC-PUFA consumption (80). Other authors claim that fish oil consumption is not dangerous in clinical populations (16) and that the empirically supported benefits of PUFA supplementation simply outweigh the yet unproven potential hemorrhagic risks (16). In humans, fish oils do not appear to decrease platelet aggregation (81) or platelet derived growth factor (82). Even when taken in conjunction with other blood thinning agents, such as aspirin and warfarin, fish oil consumption appears to have no significant effect on bleeding (83–85). Harris reviewed 19 studies involving individuals taking fish oil after percutaneous transluminal coronary angioplasty, coronary artery bypass grafting, endarterectomy or coronary angiography, and concluded that fish oil supplementation does not increase clinically significant bleeding, and concluded that PUFAs do not appear to affect bleeding (86). It should be noted however, that none of the studies reviewed, investigated the effect of bleeding in relation to cerebral ischemic or hemorrhagic stroke (86). Conversely, an earlier review by Knapp concluded that, while evidence is inconsistent, that polyunsaturated fatty acids do appear to increase template bleeding time in human populations (87). Additionally, a number of clinical studies that have investigated the effects of fatty acids after subarachnoid hemorrhagic stroke, suggest that the omega-3 fatty acid EPA inhibits vasoconstriction, while the omega-6 AA, contributes to vasospasm. Thus, in the case of subarachnoid hemorrhagic, fatty acids do appear to influence blood flow in clinical populations (64–69).

Research investigating the neuroprotective effect of *n*-3-LC-PUFAs after ischemic stroke, typically begin *n*-3-LC-PUFA administration after the surgical procedure (19, 21). In the present research, supplementation commencing after surgery was not appropriate, as we were interested in monitoring sickness behavior post-surgery, and changing the diet after surgery was likely to confound eating behaviors. However, given the increased hemorrhagic bleeding seen in the present study, we suggest that pre-feeding with *n*-3-LC-PUFA may be dangerous (16, 17, 76–80). Further research is required to clarify the potential risks of *n*-3-LC-PUFA supplementation on hemorrhagic bleeding in vulnerable populations (62, 63).

Prolonged changes and decreases in eating and drinking behaviors are a feature of sickness behaviors in animals (88). Therefore, it is not surprising that sham animals had higher food and water intake than MCAo affected animals post-surgery. As hypothesized, *n*-3-LC-PUFA supplemented animals similarly consumed more water than basal fed rats post-surgery. Our unpublished data shows non-surgery affected, normally reared, *n*-3-LC-PUFA supplemented rats, do not consume more food and water than basal diet fed rats. Therefore, it is unlikely that *n*-3-LC-PUFA fed animals drink more due to increased salt or dietary change. In the present study increased water consumption among *n*-3-LC-PUFA supplemented rats after surgery could reflect better recovery from surgery, as animals rapidly replace surgery associated blood volume loss.

Not surprisingly, MCAo affected animals showed acute motor impairment in the 5-days following surgery. However, as motor impairment scores in the first 5 days following MCAo did not correlate with infarct size as determined at 6 weeks post-surgery, indicating that early motor impairments is more likely to reflect short term MCAo related ischemia and associated edema rather than longer-term tissue atrophy. Thus, the motor impairment tests used in the present study are not appropriate measures of MCAo related cellular damage in rodents.

MCAo affected animals showed increased locomotive behaviors compared to sham operated animals, which is consistent with previous research (89). Previous authors have speculated that MCAo related locomotor deficits might arise from an inability to habituate to unfamiliar environments, possibly related to spatial mapping difficulties induced by hippocampus cell death (27–30). However, the present research does not indicate “habituation” deficits in ischemia-affected animals, as we found no difference between stroke and sham affected animals in the spatial displacement recognition test at 2, 4, and 6 weeks, post-surgery. It is possible that ischemia associated cell proliferation in the hippocampus may have already occurred by the time the first spatial displacement recognition test was conducted, at 2 weeks post-surgery, and corrected any spatial mapping disabilities that may have been previously present. Indeed, MCAo associated hippocampal neuronal degeneration is seen to peak between 12 h and 7 days after surgery, with no further degeneration after this point, in rodents (90). Additionally, neurogenesis in the adult hippocampus is seen to increase eightfold 1 week after focal ischemia, but return to basal levels 2 and 3 weeks post insult (91). After transient ischemia CA1 neurons are seen to completely repopulate by 1 month post infarct (92). After transient ischemia, treatment with growth factor restores memory function in the Morris water maze task, at 49–53 days after surgery, which positively correlates with the effect of the growth factor on the regeneration of new neurons (92).

After global ischemia, this is associated with improved spatial learning and memory functions, as assessed using a water maze (93). Alternatively, Plamondon and Khan (94) suggest that ischemia-affected rodents do not have habituation deficits at all, but rather that the testing periods often employed are not long enough to allow the animal enough time to become familiar with its environment (94).

In the present study, extensive tissue damage in the somatosensory cortex following the MCAo surgery may have contributed to the changes in locomotory behaviors, as pyramidal neurons of the second and third layers of the somatosensory cortex project to the motor cortex (29, 95). Additionally, previous research has associated amygdala damage with a decreased anxiety response that has also been associated with increased exploratory activity (96). While extensive damage was seen in the amygdala of MCAo affected animals in the present study (data not shown), the current results do not suggest that MCAo operated animals showed less anxiety-like behaviors in the free exploration test than sham operated animals. Additionally, we found a negative correlation between both the number of emergences from the hide box into the open field, the time spent moving around in the open field and the percentage of emergence time spent in the center of the open-field arena, indicating that the change in locomotion is unlikely to result from a change in anxiety-like behaviors in surgery affected animals.

We found no obvious difference in the exploration time of the novel object between basal diets fed MCAo and sham operated animals. One interpretation is that MCAo surgery does not prevent the beneficial effect of *n*-3-LC-PUFA supplementation on the exploration behavior of a novel object, or that the positive effect of *n*-3-LC-PUFAs on novel object exploration behavior may be independent of MCAo. Our findings are consistent with previous research demonstrating that anti-depressive interventions, such as electroconvulsive treatment, increase novel objects exploration, in the rodent model (97). However, it should be noted that in the present study, *post hoc* tests showed no difference between MCAo and sham operated animals, in the percentage of emergence time spent in the center of an open-field arena. The finding that MCAo operated animals explored the novel object less than sham operated animals is consistent with previous research indicating that stressful interventions (conditions of unpredictable chronic stress) induces sickness behavior and inhibit novel object exploration (98). Interestingly, genetically knocking-out the depression associated dopamine D4 receptor in mice has also been shown to be associated with less approach/explore behaviors in the novel object exploration test (44).

A limitation of the present research is that we did not investigate serum or vascular EPA and DHA levels. Previously, EPA administration post MCAo has been shown to improve local cerebral blood flow and metabolism in the rat when administered for 4 weeks via gavage (99). Serum and vasculature tissue have been shown to be more readily responsive to dietary EPA/DHA supplementation than brain tissue (100, 101). Thus it is possible that the behavioral effects of *n*-3-LC-PUFA dietary supplementation may relate to the effects of PUFA supplementation on cerebral blood flow. The present findings also indicate an increased reperfusion related hemorrhagic risk in MCAo *n*-3-LC-PUFA supplemented rats, after only a short acclimation period. Further research is required to clarify the potential risks of *n*-3-LC-PUFA supplementation on hemorrhagic bleeding in vulnerable populations. In surviving animals, *n*-3-LC-PUFA supplementation appears to reduce surgery associated hyperactive locomotor and affective symptomology.

In summary, we initially aimed to investigate effects of *n*-3-LC-PUFA supplementation on surgery induced acute and longer-term sickness behaviors, acute motor impairment and anxiety-like and locomotor behaviors. However, we were surprised to find an increased risk of hemorrhage following the reperfusion component of the surgery, in a number of MCAo *n*-3-LC-PUFA supplemented rats. While these findings appear novel in the context of experimental MCAo, they are consistent with the effect of *n*-3-LC-PUFA supplementation prior to experimental intracerebral hemorrhage and the role of omega-3 fatty acids in reducing vasoconstriction and thus increasing blood flow, after subarachnoid hemorrhage. In the present study, PUFA supplementation was not seen to influence infarct size at 6 weeks after surgery, in animals that did not experience hemorrhagic bleeding. *n*-3-LC-PUFA supplementation did decrease omega-6-fatty-acid expression, reduce sickness behaviors, acute motor impairment, and longer-term locomotor hyperactivity and depression/anxiety-like behavior. Thus, in MCAo affected rats, *n*-3-LC-PUFA supplementation appears to be associated with both beneficial and detrimental effects. The current data should be interpreted with caution as we were unable to prepare the tissue for further experimental analysis, and thus further research is required to better understand the risks of *n*-3-LC-PUFA supplementation in experimental MCAo.

## Authors Contributions

Authors’ contributions were as follows: Michaela C. Pascoe contributed to the design of the project, conducted the research, and wrote the manuscript; David W. Howells contributed to the design of the project and reviewed the manuscript; David P. Crewther contributed to the design of the project and reviewed the manuscript; Nicki Constantinou contributed to data analysis; Leeanne M. Carey contributed to the design of the project and reviewed the manuscript; Sarah S. Rewell contributed with experimental procedures; Giovanni M. Turchini contributed with experimental procedures, data analysis and reviewed the manuscript; Gunveen Kaur contributed with experimental procedures, Sheila G. Crewther contributed to the design of the project and writing and revision of the manuscript.

## Conflict of Interest Statement

The authors declare that the research was conducted in the absence of any commercial or financial relationships that could be construed as a potential conflict of interest.
